# Contextualized high-speed running and sprinting during English Premier League match-play with reference to possession, positional demands and opponent ranking

**DOI:** 10.5114/biolsport.2025.147011

**Published:** 2025-01-24

**Authors:** Ryland Morgans, Mauro Mandorino, Marco Beato, Ben Ryan, Piotr Zmijewski, Alexandre Moreira, Halil Ibrahim Ceylan, Rafael Oliveira

**Affiliations:** 1 School of Sport and Health Sciences, Cardiff Metropolitan University, Cardiff, UK; 2 Brentford FC Football Research Centre, Brentford FC, London, UK; 3 Performance and Analytics Department, Parma Calcio 1913, 43121 Parma, Italy; 4 Department of Movement, Human and Health Sciences, University of Rome “Foro Italico”, 00135 Rome, Italy; 5 School of Allied Health Sciences, University of Suffolk, Ipswich, UK; 6 Jozef Pilsudski University of Physical Education in Warsaw, 00-809 Warsaw, Poland; 7 Department of Sport, School of Physical Education and Sport, University of São Paulo, São Paulo, Brazil; 8 Faculty of Kazim Karabekir Education, Physical Education of Sports Teaching Department, Ataturk University, Erzurum, Turkey; 9 Research Centre in Sports Sciences, Health and Human Development, (CIDESD), Santarém Polytechnic University, 2040-413 Rio Maior, Portugal; 10 Santarém Polytechnic University, School of Sport, 2040-413 Rio Maior, Portugal

**Keywords:** Contextual variables, Optical tracking, Match performance, Football

## Abstract

This study aimed to compare high-speed running (HSR) and sprint distances (SD) when in- (IP) and out-of-possession (OP) during official English Premier League (EPL) match-play over three consecutive seasons considering playing position, match location and opponent ranking. Match data from 31 male professional soccer players was obtained via an optical tracking system. Results showed that during the IP phase, playing position emerged as the only significant predictor for both HSRIP and SDIP. Wingers showed the highest HSRIP compared to centre-backs who exhibited the lowest values (p < 0.001, ES = 4.19). Similar data were found for SDIP (p < 0.001, ES = 3.30). HSROP was significantly affected by both ranking difference (β = -0.039, p = 0.001) and match location (β = 0.369, p = 0.001). HSROP decreases as the ranking difference increases and tends to be higher during away matches (p = 0.001, ES = 0.24). SDOP was affected by both ranking difference (β = -0.023, p = 0.001) and match location (β = 0.166, p = 0.001) and decreased as the ranking difference increased. SDOP was also higher during away matches (p = 0.001, ES = 0.23). Additionally, a significant interaction was found between playing position and ranking difference (β = 0.005, p = 0.010). Lower ranking differences correspond to higher SDOP values. In conclusion, these findings highlight that by applying these IP and OP insights into practical coaching strategies, teams can potentially enhance individual physical performance and adaptability across different match situations and seasons.

## INTRODUCTION

Recent research has reported that English Premier League (EPL) performance demands are multifaceted with various factors influencing player physical performance. Such information may be used by practitioners to design position-specific physical and technical training [1]. Contextual factors such as location, opposition ranking, team formation, possession, and positional differences have also recently emerged as examples of crucial determinants [2–6]. Players need to be able to perform high-intensity activities, such as high-speed running (HSR; distance covered 5.5–7 m · s^−1^) and sprint distances (SD; distance covered > 7 m · s^−1^), in conjunction with other technical and tactical actions. For example, passes, shots, and tackles, and offensive and defensive transitions [7, 8]. Therefore, the context in which these high-intensity actions are performed plays a key role in optimally preparing players for positional demands and the and match-play [1, 5, 6-8]. For instance, center backs had the lowest amount of HSR and SD compared to other positions [2–8], while wide midfielders were usually the players that covered the greatest HSR distance during matches [2–8]. Thus, clearly positional play has a critical role in shaping the physical demands placed on players during match-play.

The distinct physical profiles associated with different playing positions have recently been examined [1–8]. In addition, some studies showed that central midfielders covered higher total distance (TD) at low and medium speed [9, 10] and moderate-intensity acceleration distances (distances travelled accelerating between 50–75% of an individual’s maximal accelerative capacity), when compared to attackers and defenders, among elite academy EPL players [9]. Additionally, when examining Spanish First Division [10] professional soccer players, it was reported that wide attackers and wide defenders produced the highest values of very high-speed running (5.8–6.7 m · s^−1^), and accelerations (equal to or greater than 2 m · s^−2^ during an interval time equal to or less than 0.5 seconds), and SD (> 6.7 m · s^−1^) due to the repeated attacking and defensive functions of these positions [10]. Furthermore, in a study that assessed the position-specific development of physical performance parameters over a seven-season period in the EPL, it was found that wide and forward positions increased HSR (5.5–7 m · s^−1^) and SD (> 7 m · s^−1^) more than central defenders and central midfielders [11].

Additionally, other research has reported the heightened physical demands imposed by high-ranked opponents [2, 4, 8, 11–14]. These findings highlight the need for players and teams to adapt to the varying challenges posed by quality opponents [2, 4, 8, 11–14]. Furthermore, understanding the fluctuations in physical outputs across different levels of opposition quality (low-, middle- and high-ranked teams) is pivotal for devising effective training and tactical strategies [2, 4, 8, 11–14].

Previous research demonstrated that HSR and SD have frequently been analyzed without context [15], although it is clear that context plays a key role [1–8]. For instance, previous research showed that straight-line sprinting is the most frequent action that precedes a goal-scoring opportunity during open play [16]. Therefore, future research should integrate physical and tactical factors to enhance understanding of the performance demands, as well as to integrate and contextualize training [17, 18].

Recently it was shown that other factors, such as tactical strategies, game-style and decision-making elements [14], may also play a significant role in the physical demands placed on players during elite level match-play. In contrast, when considering match locations and results, a recent study did not report any effect on physical performance during official competitions [19]. Furthermore, other tactical-contextual factors (i.e., phases of play: attacking/defensive organization and transitions) have been investigated to verify the physical effect during matches [7, 18]. Calbeck [18] found that most sprints were completed out-of-possession (OP) (58%), while in-possession (IP), “run the channel” (25%) was the most observed tactical outcome. While Bortnik et al. [7] also reported that significant effects of position were found for all analyzed metrics during transitional play (large ES; *p* < 0.001) with central attacking midfielders producing the highest TD (m · min^−1^), full-backs covering the highest SD (> 7 m · s^−1^; m · min^−1^) and wide midfielders performing the highest accelerations and decelerations (> 3 m · s^−2^; n · min^−1^) (*p* ≤ 0.05). Although these studies are some of the first to analyze the impact of tactical-contextual factors on physical parameters (i.e., sprinting), only 10 matches [7] were analyzed. Thus, limiting the generalization of the results to other teams and leagues. Therefore, further research on the impact of tactical and contextual factors on physical performance is warranted.

The concept of evaluating physical performance over an extended period, as proposed in the present study, aligns with growing body of soccer research [17, 20], emphasizing the importance of analyzing physical performance trends over multiple seasons [11, 17, 20]. This approach enables a nuanced understanding of how players adapt, evolve, and maintain physical output over time, contributing significantly to the literature on player development.

Considering the limited evidence currently available examining contextualized HSR and SD during elite-level match-play in relation to possession, players’ position and opponent ranking, the present study would seem to present real-world practical significance. Thus, the aims of this study were, firstly, to compare HSR and SD when IP and OP during official EPL match-play over three consecutive seasons; secondly, to examine any differences considering playing position, match location and opponent ranking [1–8]; and finally to analyze the influence of playing position, match location and opponent ranking when IP and OP. Based on the existing literature, the study hypothesis was that positional differences of HSR and SD while IP and OP would be evident, and match location and the opponent ranking would influence HSR and SD.

## MATERIALS AND METHODS

### Study Design

A retrospective study was conducted analyzing EPL match data from the 2021–2022 to 2023–2024 seasons for a cohort of 31 male professional soccer players. Data were collected via the Second Spectrum optical tracking system from 20 EPL stadiums.

This research utilized a three-year longitudinal study design. A non-probabilistic sampling protocol was employed to recruit the participants. The emphasis of the study was on monitoring player HSR and SD while IP and OP during competitive EPL match-play. During the observational period of seasons 2021–2022 to 2023–2024, consistent player monitoring approaches were practically implemented by club staff without any interference from the researchers [8]. These methods included all team pitch-based training sessions and all strength and power gym-based sessions per micro-cycle incorporating upper and lower body and core exercises, although these sessions were not included in the analyzes [20].

### Participants

Thirty-one professional first-team squad outfield soccer players from an EPL club were involved in the study (age 24.6 ± 5.4 years, weight 76.6 ± 6.9 kg, height 1.79 ± 0.09 m). The study team adopted a 4-3-3 or 3-5-2 formation and implemented a hybrid model of possession that included build-up play and direct-play strategies [5]. Furthermore, when OP a mixture of high-press and mid-block (a narrow and compact team shape defending the middle third of the pitch) strategies were employed.

The research inclusion criteria have previously been applied [1–5] and were: (i) named in the first-team squad at the start of all seasons, (ii) played in at least 80% of matches, (iii) only completed official team training during the study period, and (iv) completed at least 75-minutes of match-play. Additionally, the exclusion criteria for the study have also been previously employed [1–5] and included: (i) long-term injured player data, (ii) joining the team late in any of the study seasons, (iii) lack of full, complete match data, and (iv) goalkeepers, due to the different variations in the physical demands with outfield players [21].

Players were classified as: centre-backs (CB; n = 10), full-backs (FB; n = 11), centre midfielders (CM; n = 8), wingers (W; n = 5), and centre forwards (CF; n = 7). If a player fulfilled multiple playing positions during match-play, the player was categorized accordingly to each position.

All data collected resulted from normal analytical procedures regarding player monitoring over the competitive season [1, 2], nevertheless, written informed consent was obtained from all participants. The study was conducted according to the requirements of the Declaration of Helsinki and was approved by the local Ethics Committee of Cardiff Metropolitan University (Sta-9172) and the club from which the participants volunteered [22]. To ensure confidentiality, all data were anonymized prior to analysis.

### Data Collection

Data were collected in all (n = 114) regular-season EPL competitive matches played by the examined team across the three study seasons (2021–2022 to 2023–2024). League match data across the study seasons were recorded and analyzed via the optical tracking system Second Spectrum to report physical performance data. Second Spectrum has been validated by the FIFA program to meet industry standards [23]. Data were collected via semi-automated HD cameras positioned around the stadium with a sampling frequency of 25 Hz.

A total of 1351 individual match data points were examined with a median of 24.5 data points per player (range = 1–114). This resulted in 894 full or nearly full match data points for all players with a median of 17 per player (range = 1–81).

The variables analyzed were divided by the actual playing time for each player. The relative HSR (m/min, distance covered 5.5–7 m·s^−1^); and SD (m/min, distance covered > 7 m · s^−1^) were examined. The following tactical/physical variables were also quantified in this study: HSR distance when IP (HSRIP); SD when IP (SDIP); HSR distance when opposition IP (HSROP); and SD when opposition IP (SDOP).

Each match was classified based on the ranking difference between the teams at that specific point in the season. This ranking difference was treated as a continuous variable, reflecting the number of positions separating the two teams in the league standings. A positive value indicated that, at that moment in the season, the team held a higher (better) rank than its opponent, whereas a negative value signified that the team was ranked lower (worse) than its opponent. The Second Spectrum match data were processed directly using the Python programming language (Python 2.7) through the Spyder scientific development environment (https://www.spyder-ide.org/). Publishing the exact algorithms used to determine the examined measures was not possible due to the technological commercial entities keen to protect intellectual property rights [17]. Thus, the specific conversion and filtering algorithms utilized in these systems were not available.

### Statistical analysis

A two-level multilevel regression analysis was performed to assess the impact of playing position, ranking difference, and match location (home/away) on HSR and SD during IP (HSRIP, SDIP) and OP phases (HSROP, SDOP). Level 1 consisted of player positions, while Level 2 included contextual factors (ranking difference and match location). Player identity was included as a random effect to account for repeated measurements over time.

Ranking difference was treated as a continuous variable, while playing position (centre-backs = 0, full-backs = 1, centre midfielders = 2, wingers = 3, centre forwards = 4) and match location (home = 0, away = 1) were treated as ordinal variables. All independent variables were treated as fixed effects in the model, and players were treated as random effects to account for intra-player correlations due to repeated measurements.

– A stepwise approach was used to build models for each dependent variable:
– Model 1: Included the Level 1 predictor (playing position).
– Model 2: Added the Level 2 predictors (ranking difference, match location).
– Model 3: Included the interaction between playing position and ranking difference.
– Model 4: Included the interaction between playing position and match location.
– Model 5: Included the interaction between ranking difference and match location.

Prior to conducting the multilevel model analysis, assumptions of normality and homogeneity of variance were assessed for all variables. Normality was examined using QQ plots, while homogeneity of variance was evaluated with Bartlett’s test to ensure the appropriateness of the model assumptions. The model’s goodness-of-fit was assessed using the Akaike Information Criterion (AIC), with lower AIC values indicating a better fit. When statistically significant differences were observed in the multilevel regression analysis, the least significant difference approach to multiple comparisons was adopted, as suggested [24]. Standardized effect sizes, defined as the mean difference to the pooled standard deviation, were also calculated. Effect size (ES) values of > 0.2 (small), > 0.5 (medium), and > 0.8 (large) were interpreted as small, moderate, and large differences, respectively [25]. Statistical analyzes were performed using IBM SPSS Statistics, version 28.0 (IBM Corp., Armonk, NY, USA). The threshold for statistical significance was set at *p* < 0.05.

## RESULTS

**FIG. 1:**
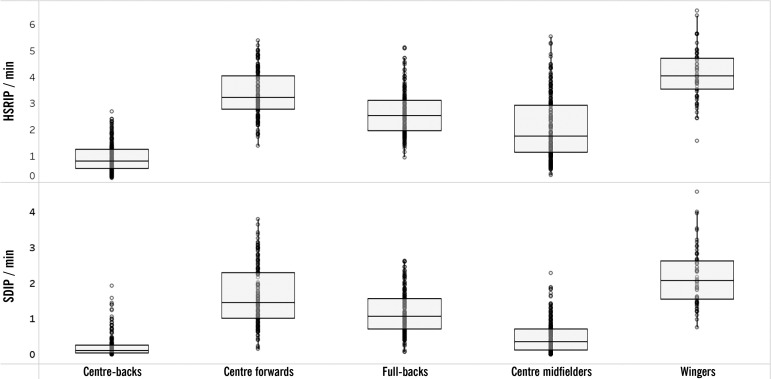
Comparison of High-Speed Running (HSR) and Sprint Distance (SD) per minute during the in-possession phase (IP) across the different playing positions.

The results of the multilevel regression analysis are summarized in Table 1, presenting β, *p*, and AIC values. Box plots were used to represent the distribution, central tendency (median), and spread (interquartile range) of HSRIP, SDIP, HSROP, and SDOP according to playing position and match location. The results showed that during the IP phase, playing position (Model 1) emerged as the only significant predictor for both HSRIP and SDIP. Figure 1 illustrates the variation in HSRIP and SDIP across different playing positions, high-lighting the positional differences in physical performance. Wingers showed the highest HSRIP compared to centre-backs who exhibited the lowest value (*p* < 0.001, ES = 4.19). Similar data were found for SDIP (*p* < 0.001, ES = 3.30).

**TABLE 1:** Multilevel regression analysis of High-Speed running per minute (HSRIP, HSROP) and Sprint distance (SDIP, SDOP) according to playing position, ranking difference, match location, and their interaction.

High Speed Running	HSRIP / min					HSROP / min				

	Model 1	Model 2	Model 3	Model 4	Model 5	Model 1	Model 2	Model 3	Model 4	Model 5

Fixed Variables										
Playing position	0.277 (0.001)	0.286 (0.001)	0.293 (0.001)	0.284 (0.001)	0.285 (0.001)	0.122 (0.149)	0.100 (0.223)	0.111 (0.178)	0.119 (0.172)	0.119 (0.171)

Ranking difference	N.E.	0.011 (0.003)	0.009 (0.124)	0.009 (0.120)	0.012 (0.090)	N.E.	-0.039 (0.001)	-0.046 (0.001)	-0.046 (0.001)	-0.049 (0.001)

Match location	N.E.	-0.042 (0.379)	-0.042 (0.381)	-0.063 (0.386)	-0.073 (0.326)	N.E.	0.369 (0.001)	0.369 (0.001)	0.391 (0.001)	0.403 (0.001)

Playing position × Ranking difference	N.E.	N.E.	0.001 (0.572)	0.001 (0.589)	0.001 (0.609)	N.E.	N.E.	0.004 (0.269)	0.004 (0.262)	0.004 (0.252)

Playing position × match location	N.E.	N.E.	N.E.	0.013 (0.703)	0.014 (0.682)	N.E.	N.E.	N.E.	-0.013 (0.774)	-0.014 (0.754)

Ranking difference × match location	N.E.	N.E.	N.E.	N.E.	-0.005 (0.463)	N.E.	N.E.	N.E.	N.E.	0.006 (0.527)

Goodness of fit (AIC)	**1890.973**	1895.174	1904.830	1909.592	1916.967	2479.547	**2415.036**	2423.140	2427.319	2434.188
**Sprint Distance**	**SDIP / min**					**SDOP / min**				

	**Model 1**	**Model 2**	**Model 3**	**Model 4**	**Model 5**	**Model 1**	**Model 2**	**Model 3**	**Model 4**	**Model 5**

**Fixed Variables**										

Playing position	0.199 (0.001)	0.195 (0.001)	0.190 (0.001)	0.183 (0.001)	0.184 (0.001)	-0.065 (0.123)	-0.070 (0.102)	-0.056 (0.189)	-0.060 (0.187)	-0.060 (0.184)

Ranking difference	N.E.	-0.002 (0.338)	-0.001 (0.996)	0.001 (0.983)	0.003 (0.443)	N.E.	-0.014 (0.001)	-0.023 (0.001)	-0.023 (0.001)	-0.020 (0.001)

Match location	N.E.	0.031(0.295)	0.031 (0.299)	0.014 (0.751)	0.003 (0.934)	N.E.	0.164 (0.001)	0.166 (0.001)	0.154 (0.009)	0.146 (0.015)

Playing position × Ranking difference	N.E.	N.E.	-0.001 (0.398)	-0.001 (0.382)	-0.001 (0.356)	N.E.	N.E.	0.005 (0.010)	0.005 (0.010)	0.005 (0.011)

Playing position × match location	N.E.	N.E.	N.E.	0.010 (0.615)	0.011 (0.580)	N.E.	N.E.	N.E.	0.007 (0.801)	0.007 (0.776)

Ranking difference × match location	N.E.	N.E.	N.E.	N.E.	-0.006 (0.191)	N.E.	N.E.	N.E.	N.E.	-0.004 (0.438)

Goodness of fit (AIC)	**1107.383**	1120.790	1130.982	1136.569	1143.708	1534.874	1516.019	**1513.688**	1522.957	1530.698

**FIG. 2:**
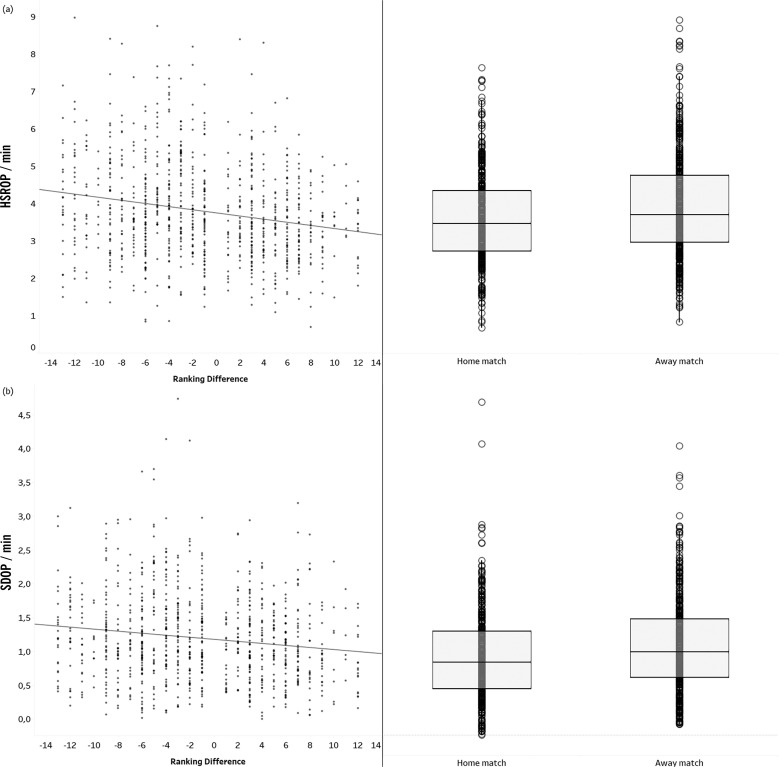
Comparison of High-Speed Running (HSR) and Sprint Distance (SD) per minute during the out-of-possession phase (OP) according to contextual factors: relationship between the ranking difference and comparison between home and away matches.

In contrast, contextual factors had a greater influence during the OP phase. Specifically, HSROP was significantly affected by both ranking difference (β = -0.039, *p* = 0.001) and match location (β = 0.369, *p* = 0.001), as depicted in Figure 2. These results suggest that HSROP decreases as the ranking difference increases and tends to be higher during away matches (*p* = 0.001, ES = 0.24).

**FIG. 3:**
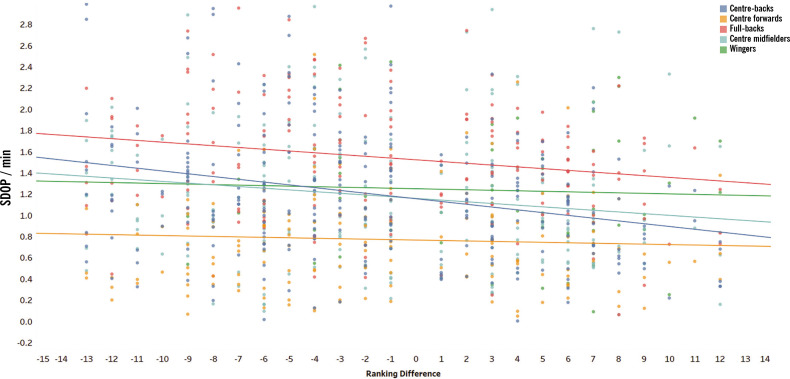
The effect of interaction between playing position and ranking difference on Sprint Distance (SD) per minute during the out-of-possession phase (SDOP).

Similar patterns were observed for SDOP, that was affected by both ranking difference (β = -0.023, *p* = 0.001) and match location (β = 0.166, *p* = 0.001), as presented in Figure 2. Further, SDOP decreases as the ranking difference increases and tends to be higher during away matches (*p* = 0.001, ES = 0.23). Additionally, a significant interaction was found between playing position and ranking difference (β = 0.005, *p* = 0.010). As shown in Figure 3, lower ranking differences correspond to higher SDOP values. The slope for each position highlights the varying impact of ranking difference: centre-backs (slope = -0.026), full-backs (slope = -0.016), centre midfielders (slope = -0.016), wingers (slope = -0.001), and centre forwards (slope = -0.001).

## DISCUSSION

The aims of this study were: to compare HSR and SD when IP and OP during official EPL match-play over three consecutive seasons; and to examine any differences considering playing positions, match location and opponent ranking. The main finding of this study is the clear distinction between the factors influencing physical performance during IP and OP phases. Specifically, the IP phase was primarily affected by the player’s position, whereas the OP phase was influenced by contextual factors such as ranking difference and match location. This highlights the importance of separating these two phases when analyzing physical performance and highlights the need to contextualize HSR and SD profiles in relation to broader match dynamics.

The results of this study reveal critical insights into the physical performance dynamics in elite EPL soccer, particularly distinguishing between phases of play notably IP and OP. During the IP phase, playing position emerged as a significant predictor for both HSRIP and SDIP. This finding reinforces existing literature that suggests player tactical roles significantly dictate movement patterns, and ultimately distances and speeds, during IP [26]. For instance, wingers in the EPL typically engage in more fast, explosive actions, such as sprinting into space or making sharp cuts to receive passes, to exploit gaps in the defense when IP, maximizing HSR and SD to create goal-scoring opportunities [18]. These notions have previously been reported [26]. For example, in the Chinese Super League, when the team with higher possession attacks, three lines of players move together deep into the opponents’ half which produces greater distances to cover, specifically for wingers [26]. Furthermore, when the higher possession team needs to defend, wingers must sprint back to mark an opponent player or chase/press the ball until it is regained, which can further result in a fast counterattack [26]. In contrast, defenders and midfielders focus more on maintaining team structure and possession retention [27, 28]. For instance, a centre midfielder may prioritize positioning to intercept passes or transition play, which typically results in less HSR compared to forwards although is crucial for overall team shape and strategy when IP and OP [27, 28].

Conversely, during the OP phase analysis, contextual factors played a more pronounced role in influencing physical performance metrics. The negative relationship between ranking difference and HSROP suggests that as the disparity in team rankings increases, players may reduce high-intensity efforts. This result aligns with findings from previous studies indicating that players tend to adjust physical performance based on perceived competitive pressure [12]. For example, a mid-table team facing a top-tier opponent might conserve energy by reducing high-intensity runs, especially if prolonged periods of defending, OP are anticipated [8]. This can lead to less effective pressing and increased vulnerability. Notwithstanding, previous research conducted in the Persian Gulf Premier League indicated that soccer players should be physically prepared to cover a relatively higher TD when playing against superior opponents [29].

Additionally, the positive impact of match location on HSROP highlights the importance of home advantage. These findings support recent research that reported external match load variables were influenced by several contextual factors including match location which impacted TD, HSR and SD when playing home or away against top, middle or bottom six teams [4]. Furthermore, teams playing at home have often experienced increased motivation, leading to enhanced physical performance [27]. This home location may create an intimidating atmosphere for visiting teams, often pushing players to exert more effort during home matches [13]. Nonetheless, the context of the league could be of major importance, since previous research in the Persian Gulf Premier League showed no difference regarding HSR or SD when playing at home versus playing away [30].

The observed patterns for SDOP mirrored those of HSROP, further illustrating the impact of contextual factors such as possession. The significant interaction between playing position and ranking difference emphasizes the complexity of these dynamics. The variation for each position indicates altering sensitivities to ranking differences, with CB showing the most significant negative slope. This finding suggests that defenders may prioritize defensive responsibilities over high-intensity efforts when faced with stronger opponents which supports previous research [26]. For example, a defender may focus on tactical positioning against an elite-ranked forward, rather than on performing HSR and SD, especially if the attacking team has a significant ranking advantage [31]. However, this may be unavoidable based on the opposition forward’s physical profile and tactical role. In contrast, the minimal slopes for centre forwards and wingers suggest that sprinting profiles may remain more consistent regardless of the ranking context. However, fast, explosive players typically known for speed and offensive actions, will continue to execute high-intensity runs to stretch the defensive line, highlighting the high physical demands of these tactical roles [28]. As previously suggested, this may frequently occur as a positional requirement for wingers [26].

These results underscore the key interaction between player role and contextual variables that influence physical performance in elite soccer. Understanding these dynamics can inform coaching strategies, particularly in tailoring training programs and match preparations that account for both the tactical requirements of specific positions and the external competitive factors. For instance, coaches might implement specific drills that simulate match conditions based on the oppositions strengths, helping players adapt intensity levels accordingly [32]. By addressing these contextual nuances, teams can enhance physical performance in various match situations, potentially leading to improved team success.

### Practical Applications

The findings of this study offer several practical applications for coaches, analysts, and sports scientists involved in elite soccer, particularly in the EPL. For instance, previous research has stated that HSR and SD may be utilized to examine perceived recovery [33] and the present findings align with such load management theory, suggesting that contextualized HSR and SD can provide enhanced information regarding fatigue monitoring frameworks to optimize player recovery. Potentially key applications based on the distinction between physical performance factors during IP and OP phases may include tailored training programs where coaches can design specific training regime that address the unique demands of each playing position during different phases. For example, centre forwards and wingers may emphasize drills that enhance explosive speed, agility, and sharp cuts to improve HSR and SD when IP. This could include various-sided games (with large pitch sizes) where players must make quick transitions from defending to receiving the ball to create attacking positions, while defenders and midfielders may focus on tactical awareness and positioning drills, incorporating scenarios that simulate intercepting passes and maintaining defensive shape during OP phases. This may involve practicing positioning against different attacking styles to ensure players are prepared for various match contexts. For example, simulated drills focusing on rapid OP transitions could be implemented to enhance SD during defensive phases. Additionally, small-sided games of 4 versus 4 (or a lower number) [34], with man marking [35] and reduced number of ball touches [36] may contribute to a greater tactical component and depending on the intensity, duration and rest periods of the drill, may provide an aerobic or anaerobic stimulus. A potential advantage of delivering position-specific drills over varied sided games is related to the ehnaced focus on player physical fitness that cannot always achieve high physical demands during these types of training games.

Additionally, match preparation strategies where understanding the influence of ranking differences and match location can inform tactical preparations such as analyzing opponent strengths. Coaches may develop strategies that account for the opponent’s ranking, adjusting the team’s pressing intensity. For instance, against a top-ranked team, a mid-table team might opt for a more compact formation to conserve energy and counter-attack effectively rather than pressing high up the pitch and leaving space behind the defensive line that may be exploited. Finally, contextualized player profiles can be created that reflect individual roles and contextual influences such as role-specific metrics that assess players based on the specific tactical role assigned. For example, measuring a midfielder’s success in transitions can help in evaluating contributions more accurately.

### Limitations and Future Research Direction

While this study highlights the contextualized high-intensity match actions of elite soccer players with reference to possession, positional demands and opponent ranking, it is essential to acknowledge certain limitations. Factors such as individual player characteristics and opposition team strategies may influence these patterns and thus must be considered the principal limitation associated with the current study. However, such characteristics and context are almost impossible to include in the practice of soccer science research. In addition, the analyzed data came from one EPL team and thus must be interpreted with caution. Future research could therefore consider individual player profiles and other contextual factors (e.g., match outcome) [4] to provide a more comprehensive understanding of the intricacies involved in player physical performance against varying levels of opposition and identify any further positional differences. Future research could also explore differing tactical systems since other studies have highlighted these influence match load [7, 8, 14]. Additionally, future studies should consider how tactical strategies, such as high pressing or defensive blocks, amplify or mitigate the influence of ranking difference and match location on HSR and SD metrics. Furthermore, future research could explore the inclusion of tactical factors (e.g., field tilt, territorial domination) to further understand how match effort and high-speed demands are influenced by different playing styles. Another suggestion is the inclusion of different teams (e.g., from different countries such France [5], Spain or Germany) with different tactical philosophies or competing in the same league but with distinct physical demands. Finally, the analysis of accelerations and decelerations [4, 6] with the same study design would provide additional insight for coaches, analysts, and sports scientists involved in elite soccer.

## CONCLUSIONS

This study identified distinct factors influencing physical performance during IP and OP phases. Specifically, the IP phase was primarily influenced by the player’s position, while the OP phase was more sensitive to contextual factors such as ranking difference and match location. During the IP phase, playing position emerged as the only significant predictor for both HSRIP and SDIP, with wingers displaying the highest HSRIP, in contrast to centre-backs, who showed the lowest values.

In conclusion, these findings highlight that by applying these insights into practical coaching strategies, teams can potentially enhance physical performance and adaptability across different match situations and seasons. This comprehensive understanding of how player positions and contextual factors interact during both IP and OP phases will not only improve individual player physical performance but may also contribute to overall team success.
